# Ten Ways to Improve the Use of Statistical Mediation Analysis in the Practice of Child and Adolescent Treatment Research

**DOI:** 10.1007/s10567-012-0114-y

**Published:** 2012-03-15

**Authors:** Marija Maric, Reinout W. Wiers, Pier J. M. Prins

**Affiliations:** Department of Developmental Psychology, University of Amsterdam, Weesperplein 4, 1018 XA Amsterdam, The Netherlands

**Keywords:** Statistical mediation analysis, Treatment outcome, Youth, Ladder of mediation evidence

## Abstract

Despite guidelines and repeated calls from the literature, statistical mediation analysis in youth treatment outcome research is rare. Even more concerning is that many studies that *have* reported mediation analyses do not fulfill basic requirements for mediation analysis, providing inconclusive data and clinical implications. As a result, after more than five decades of research, it is still largely unknown through which processes youth treatment works and what the effective treatment components are. In this article, we present ten ways in which the use of statistical mediation analysis in youth treatment outcome research may be improved. These ten ways are related both to conceptual and methodological issues. In discussing how youth clinical researchers may optimally implement these directions, we argue that studies should employ the strongest research designs possible. In so doing, we describe different levels of a mediation evidence ladder. Studies on each step of the ladder contribute to an understanding of mediation processes, but the strongest evidence for mediation is provided by studies that can be classified at the highest level. With the help of the ladder of mediation evidence, results from youth mediation treatment outcome research can be evaluated on their scientific as well as clinical impact.

Mediation analysis is an important tool for (clinical) researchers because it explains *why* or *how* a certain treatment achieves its effects. Treatment mediators are ‘mechanisms or processes through which a treatment might achieve its effects’ (Kraemer et al. 2002, p. 878). For example:

- Does anxious self-talk mediate treatment outcomes for youth diagnosed with an anxiety disorder (Kendall and Treadwell 2007)?
- Does active treatment condition cause levels of parent management skills to increase and deviant peer associations to decrease, and does this lead to a decrease in antisocial youth behaviors (Eddy and Chamberlain 2000)?
- Do treatment acceptance and session attendance mediate treatment outcomes in families with children with ADHD (MTA Cooperative Group 1999)?

Identification of these mechanisms can improve youth treatments by identifying effective treatment components, and the costs of the treatments can be reduced by removing ineffective treatment components. Importantly, through investigations of mediators of youth treatment outcomes, the dissemination of treatments that work to clinical practice can be facilitated. A graphic representation of the role that mediators are likely to play in treatment for youth disorders is presented in Fig. 1. It is important to distinguish between treatment mediators and moderators (Holmbeck 1997; Kraemer et al. 2002). Treatment moderators are ‘pretreatment or baseline variables that identify subgroups of patients within the population who have different effect sizes’ (Kraemer et al. 2007, p. 1286). As such, moderators of treatment outcome help to answer a different type of question; that is, *for whom* is treatment effective, and *for whom* is it less effective?

**Fig. 1:**
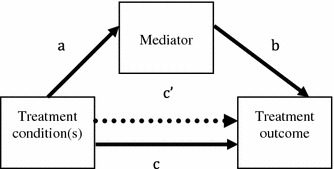
A single mediation model exemplifying mediation of treatment outcome

Within the youth treatment outcome literature, the importance of identifying mediators was expressed more than a decade ago (e.g., Holmbeck 1997) and continues today (e.g., Kendall 2009). Further, important directions for the study of mediators of youth treatment outcomes were provided (e.g., Kazdin and Nock 2003; Prins and Ollendick 2003; Weersing and Weisz 2002). Yet, the number of mediation studies conducted with youth populations is relatively small. For example, in their review of mediators of youth psychotherapies, Weersing and Weisz stated a decade ago that only two treatment studies for internalizing disorders in youth (Kolko et al. 2000; Treadwell and Kendall 1996) had attempted to investigate mediators of treatment outcome. Since then, to our knowledge, four other studies on internalizing disorders including mediation analyses have been reported (Alfano et al. 2009; Kaufman et al. 2005; Kendall and Treadwell 2007; Lau et al. 2010). It is not the case that the amount of treatment outcome research has decreased since then. To the contrary, several new treatment outcome studies for youth with internalizing disorders have been conducted (e.g., Bodden et al. 2008; Bögels and Siqueland 2006; Chorpita et al. 2004; Hudson et al. 2009; Liber et al. 2008), but these studies have not reported analyses of mediators of treatment outcomes.

Even more concerning is that the research designs of many studies that *have* reported mediation analyses do not fulfill the most established requirements for mediation analysis (MacKinnon 2008). Consequently, the evidence for the mediating effects in these studies is weak, and implications for research and clinical practice therefore inconclusive. For example, four of the above-mentioned studies (Alfano et al. 2009; Kaufman et al. 2005; Kendall and Treadwell 2007; Treadwell and Kendall 1996) used only a pre-post design, and, therefore, did not fulfill the temporal precedence requirement of mediation. Alfano et al. (2009) used a single-condition design (i.e., without a treatment control condition). As will be made clear, the absence of conditions of temporal precedence and of a control condition provides weak evidence for mediation. Clearly, despite many advantages related to studying mediators of youth treatment outcomes, this topic is still largely being neglected or inadequately addressed in the field of youth internalizing disorders.

In the area of youth externalizing disorders, several studies with more sophisticated mediation designs can be found. For example, using multisource and multidomain assessments performed at 4-assessment points in a large sample of 579 children with ADHD, the MTA group (1999) tested treatment acceptance and attendance as mediators of three treatment conditions (medication management, behavioral treatment, community care). Further, there are attempts to use innovative statistical techniques to study mediation (e.g., Henggeler et al. 2009). Yet, challenging conditions are being reported such as samples too small for the data-analytic techniques used to test for mediation (e.g., Eddy and Chamberlain 2000), and the use of pre-post designs only (e.g., Nock and Kazdin 2005). At this moment, what is unknown about processes through which treatments for externalizing youth work outweighs what is already known.

This article presents a guide to important ways in which the use of mediation analyses in child and adolescent treatment outcome research can be improved. First, we discuss ten ways related to conceptual and methodological issues regarding mediation analysis through which this goal can be accomplished. Second, based on a discussion of research designs, we place different designs on a ‘scientific ladder of mediation evidence’, illustrating which designs provide strong or weak evidence for mediation relations. Finally, we urge both clinical researchers to conduct mediation analyses in their studies, and journal editors and reviewers to demand these analyses when reviewing journal articles. This joint effort will help us understand better how youth treatments work and will improve the efficacy and effectiveness of these treatments.

## Ten Ways to Improve the Use of Statistical Mediation Analysis in Youth Treatment Outcome Research

### Specifying and Choosing the Mediators of Treatment Outcomes

The very first question when planning to design a mediation study of treatment outcome is ‘which mediator or mediators are going to be tested?’ In most cases, theory provides the basis for the mediators to be investigated. As the treatment is designed to produce changes in certain disorder-related symptoms, the researchers should consider which variables are to be targeted. For example, the theoretical underpinning of cognitive-behavioral therapy (CBT) is that distorted cognitive processing is involved in the cause and maintenance of anxiety (e.g., Stallard 2009) and depression in youth (e.g., Abela and Hankin 2008), and for change to occur, change in cognitions should happen. Mostly, strategies such as cognitive restructuring are implemented to alter young persons’ distorted thinking. In line with this theory, several studies have investigated cognitions as a mediator of treatment outcomes. Kendall and Treadwell (2007), Treadwell and Kendall (1996), and Kaufman et al. (2005), for example, found evidence for negative cognitions being a mediator of CBT outcomes for youth anxiety and depression, while Kolko et al. (2000) did not. Examples of potential mediators from other theories are also imaginable. A frequently tested theory in the field of treatments for externalizing disorders in youth is the coercion theory (Patterson et al. 1992), which proposes that conduct behavior is maintained through poor family management skills of supervision, discipline and positive rewards. Several studies tested these parenting practices as mediators of treatment outcome (Eddy and Chamberlain 2000; Fossum et al. 2009; Gardner et al. 2006; Hagen et al. 2011). Indeed, evidence was found that these parent management trainings improved parenting skills which, in turn, led to less child problem behaviors.

Besides using treatment theory, results from previous research may inform the choice of potential mediators (MacKinnon 2008). The review of de Boo and Prins (2007) represents a good example of recommendations on candidate mediators, based on an extensive review of the theory underlying treatments for youth ADHD and the available empirical evidence. In the absence of empirical evidence, common sense and focus groups may be used (MacKinnon 2008). Using common sense, researchers can identify what seems to be the best target for the treatment. A focus group discussion is a qualitative way of gathering information regarding potential mediators (e.g., asking: ‘What has helped you the most in controlling your anger?’). It is recommended that, whenever possible, potential mediators are chosen based on theory and previous empirical research. In the absence of these two, common sense and focus groups methods can be used to generate hypotheses on potential mediators. Ideally, mediators identified based on common sense and focus groups may be tested in single-case experiments, prior to their investigations in randomized clinical trials.

With regard to these different methods of selecting mediators, it should be noted that particularly in the field of child and adolescent treatment, we should be thoughtful of choosing potential mediators that are related to child (e.g., treatment adherence), parental (e.g., discipline), and familial (e.g., family cohesion) functioning. In some cases (e.g., school refusal), it is also useful to investigate the functioning of school-practitioners as this may influence school return. Further, changes in (neuro)biological indicators of functioning (e.g., brain functioning, hormonal changes, sleep and eating patterns) should also be considered as potential mediators. It is important to give attention to all these different mediators because they can, either apart from each other or simultaneously, lead to successful treatment outcome.

Clinical researchers should also consider testing both specific and non-specific processes as mediators of treatment outcomes. Specific processes refer to the processes aimed to be changed by an active treatment (e.g., avoidance behavior and dysfunctional thoughts in CBT). Non-specific processes refer to characteristics that are shared by most treatments and include, for example, the therapeutic alliance and therapist’s competence and adherence to the treatment protocol (Chatoor and Krupnick 2001).

Finally, given the controversial findings showing that some treatments may actually worsen complaints rather than decrease them (e.g., Dishion et al. 1999; Macgowan and Wagner 2005), we should also be sensitive to capture iatrogenic mediators of youth treatment outcomes. For example, Dishion et al. (1996) found that group deviancy training predicted future deviant behavior in adolescence. Group processes were suggested to account for these iatrogenic effects, but formal mediation tests of these processes were not conducted. More recently, Mager et al. (2005) investigated this hypothesis in a clinical trial comparing the effectiveness of problem-solving skills training in a ‘pure’ group of adolescents (all members with conduct problems) with a ‘mixed’ group (adolescents with and without conduct problems). The deviancy training hypothesis was not confirmed, in as much as youth assigned to the ‘pure’ group had higher rates of positive in-session behavior (e.g., complementing others) and lower rates of negative behavior (i.e., not following directions) than the ‘mixed’ group of youths. Moreover, it was shown that the deviancy training in the ‘mixed’ group condition accounted for more externalizing behavior at post-treatment. As the discussion regarding benefits of youth group therapy continues (e.g., Van Manen et al. 2005; Weiss et al. 2005), focus on mediators of therapy outcomes can provide a fine-graded analysis of group processes that may lead to iatrogenic effects and to positive treatment outcomes, and also whether these effects are being moderated by factors such as personality of group members.

### Investigating Potential *Non*-mediators

According to ‘mediation theory’, the treatment should produce change in the constructs it was designed to change (e.g., negative cognitions, ineffective parental discipline) and should not influence constructs it was not designed to change. This notion can be tested through an investigation of the effects of the, so-called, potential *non*-mediators. These are variables expected not to be affected by the treatment. If the mediating effect is stronger for the proposed mediator than for the proposed non-mediator, this is additional evidence for the mediation relation. For example, an intervention is designed to reduce social anxiety and the proposed mediator is teaching clients to become more socially skilled. A potential non-mediator is a report of happiness during treatment. The evidence for mediation should be stronger for the social skills mediator than for the happiness mediator, if an increase in social skills leads to a decrease in social anxiety.

Another potential non-mediator may be a variable that is expected to be changed by another treatment. This idea is illustrated in two clinical trials comparing the efficacy of CBT and medication treatment (Segal et al. 2006), and mindfulness-based cognitive therapy (MBCT) and medication treatment (Kuyken et al. 2010) for adult recurrent depression. Kuyken et al. (2010) investigated potential mediators of MBCT outcomes. Compared to medication treatment, MBCT was associated with greater levels of cognitive reactivity (i.e., reactivated network of distressing thoughts and feelings) post-treatment, but this did not mediate poorer treatment outcome (more depression). While MBCT evoked distressing thoughts and feelings, the patients were also learned to attenuate them with self-compassion without trying to change them (moderating role of self-compassion). In the maintenance antidepressants (mADM) group, greater reactivity predicted worse outcomes: relapse and depressive symptoms. Although Segal et al. (2006) did not formally conduct tests of mediation, they found that less cognitive reactivity was associated with the CBT group relative to the mADM group. The findings from these two studies suggest that cognitive reactivity is not a mediator of MBCT outcome, and that the two active treatments for recurrent depression (MBCT and CBT) probably work through different mechanisms.

### Optimizing the Assessment of Mediators

Using inadequate measures of mediating variables can result in the absence of mediating effects even when the proposed mediator *is* actually a mediator of treatment outcome, and even when the treatment program *is* actually able to significantly change the potential mediator. Valid and reliable assessment of the mediators is of crucial importance for the correct identification of the mediators of youth treatment outcomes. As Hoyle and Kenny (1999) found, unreliable assessment of the mediators can have negative effects on statistical power and Type I errors, and, in that case, the findings from mediation analyses should be interpreted with caution. Though some statistical techniques (e.g., structural equation modeling) can to some extent compensate for the unreliability in the measurement of the mediator, these techniques require rather large sample sizes (i.e., ≥100; Hoyle and Kenny 1999), which is perhaps one of the biggest challenges in youth treatment outcome research. However, as validity and reliability of measurements are often associated with the amount of implemented measures and/or items, it should be noted that sometimes practical constraints (i.e., burden for the clients, financial costs) will influence the choice and amount of assessment. Nevertheless, finding and selecting instruments with good psychometric properties has enormous benefits, and clinical researchers should choose instruments with excellent properties in youth treatment outcome studies.

With regard to reliability, in the case of investigating multiple mediators, it would be useful to measure each mediator with approximately the same amount of measures and/or items because item number can influence the reliability of the measures, and therefore, the psychometric properties can differ per mediator (Kaufman et al. 2005). To enhance the validity of a mediation study, several issues are of relevance. First, multiple indicators of mediating variables should be used. In general, most child and adolescent studies have used single indicators of potential mediators, being assessed from either the perspective of the child (e.g., Kendall and Treadwell 2007) or the parents (e.g., Henggeler et al. 2009). The study by Tein et al. (2004) presents an example of assessing mediators from the perspectives of multiple informants. Tein et al. assessed positive parenting (e.g., discipline, communication) from the perspectives of both child and caregiver to investigate parent-related mediators of a family bereavement treatment program. It should be noted that in case of multiple informants, an additional challenge is how to deal with informant discrepancies. There is an ongoing discussion whether informant discrepancies are a source of measurement error or whether they represent meaningful information on youth psychopathology. Each perspective suggests a different approach to handling informant discrepancies (i.e., use of composite scores vs. algorithms). This issue is beyond the scope of this article, for more information see Achenbach (2011) and De Los Reyes (2011).

A second issue related to the validity of a mediation study includes the use of multiple methods of assessment. To assess potential mediators, youth treatment outcome research has until now been oriented toward the use of self-reports (e.g., Beauchaine et al. 2005; Kaufman et al. 2005; Kazdin and Wassell 1999; Kendall and Treadwell 2007; Kolko et al. 2000; Stice et al. 2007; Treadwell and Kendall 1996). Several other assessment methods can be informative when testing mediators. Besides self-reports, qualitative data gathered post-treatment (e.g., asking young clients ‘What was most helpful for you in preparing to face a feared social situation?’) can provide unique insights into the treatment process (Dworkin et al. 2006; MacKinnon 2008). Further, implicit processes that occur outside conscious control and awareness may also play a role in youth psychopathology (Huijding et al. 2010), but little has been done with respect to implicit measurement of potential mediators of treatment outcomes in youth. With adults it has been shown that change in implicit panic associations was a significant predictor of change in panic symptom severity over the course of time (Teachman et al. 2008). Recently, neurobiological foundations of psychotherapy protocols have become a topic of study. Linden (2006) summarized adult studies that investigated changes in brain processes assessed with functional magnetic resonance imaging (fMRI) following psychological treatments for anxiety and depression. Brain activation processes may also be assessed during youth treatments and investigated as mediators of treatment outcomes. Neuroimaging techniques are then especially helpful because there is evidence of definite brain changes during adolescence (Crone 2009), and it would be interesting to know whether these changes are due to environmental influences such as receiving a particular treatment. These techniques can then be utilized to measure brain processes frequently on several assessment points during and after the treatment.

### Temporal Precedence

When establishing mediation of treatment outcome, one wants to know whether treatment affects the mediator and whether these changes in the mediator lead to changes in treatment outcome (Kraemer et al. 2002; MacKinnon 2008). This sequence of events involves an aspect of temporality. That is, to establish mediation, changes in the mediating variable should follow administration of the treatment and should precede changes in the treatment outcome (Kraemer et al. 2002; MacKinnon 2008). To investigate this hypothesis, three aspects of a mediation study should be met. First, the study design should incorporate more than two assessment points. Second, the measures of all variables (mediators and treatment outcomes) should be taken at all assessment points. This provides an opportunity to test for the reciprocity of mediating effects (i.e., changes in cognition lead to changes in anxiety behaviors and not vice versa). The third aspect involves the notion that assessments should be conducted at the moments when changes in the mediator are expected to cause changes in the treatment outcome. The first two aspects are more practical involving some issues that need to be resolved with regard to, for example, time (e.g., how many assessment points), and cost constraints (e.g., researcher time associated with the assessment). Most mediation studies with youth with internalizing and externalizing disorders have tested for mediation with designs incorporating only two, pre- and post-treatment (e.g., Kendall and Treadwell 2007; Nock and Kazdin 2005) or three, pre-, post-, and follow-up (e.g., Kazdin and Wassell 1999; Leve and Chamberlain 2007), treatment assessment points.

The third aspect is probably the most challenging one to address as hypotheses need to be made with regard to the assessment points (during and after treatment) at which the mediator will change and at which these changes will lead to changes in the treatment outcome. Ideally, mediator processes should be captured at whatever time point maximum change in the treatment outcome is assumed; this could be early in treatment, at mid-treatment, but also later in treatment. Previously, it has been suggested for adult internalizing disorders that most changes happen early in treatment (e.g., DeRubeis et al. 1990; Strunk et al. 2010) and, therefore, mediators should be assessed regularly in the beginning and throughout treatment. On the other hand, Hagen et al. (2011) have found mediating effects in the final phase of treatment. Parent management training led to greater effective discipline at post-treatment which led to lower child aggression at 1-year follow-up.

Perhaps the best chance to capture mediating processes is to include an assessment of mediators as frequently as possible, at least before, during and after treatment. An example of a youth treatment outcome study in which attempts were made to test mediators frequently is a study by Kolko et al. (2000) on the efficacy of treatments for youth depression. Potential mediators (i.e., cognitive distortions, hopelessness, family dysfunction) were assessed at pre-, during, post-treatment, and at five follow-up time points. Another example is a study on the efficacy of multidimensional treatment foster care in youth with antisocial problems. Eddy and Chamberlain (2000) assessed potential mediators (i.e., discipline, supervision, and adult-youth relationship) at six time points: pre-treatment, 3-months post-, and 6-, 12-, 18-, 24-months follow-up. An even stronger test of temporal precedence would be facilitated via a design in which there are more assessment points during the active phase of the treatment (DeRubeis et al. 1990; DeRubeis and Feeley 1990; Moscovitch et al. 2005; Stice et al. 2010). For example, in their study on the efficacy of multisystemic therapy for adolescents with externalizing problems, Deković et al. (2011) assessed parental mediators on 7 occasions; at pre- and post-treatment, and at five monthly within-treatment assessment points. Another suggestion for assessing candidate mediators during treatment is directly after the introduction of the treatment component hypothesized to produce changes in the mediator (i.e., assess negative cognitions after the cognitive therapy component).

Probably the most rigorous test of temporal precedence is to include assessment of the mediators and treatment outcome variables on a session-by-session basis (Moscovitch et al. 2005) or on a day by day-level (Polman et al. 2010). With regard to youth, there are indications that session-based assessments may impair the motivation to participate in treatment (Stice et al. 2010). The amount of assessment that can be administered on a session or daily basis in youth treatment outcome studies therefore may be limited. A recent initiative by Weisz et al. (2011) provides a possible solution for this challenge. The authors developed a very short, Youth Top Problems measure, an idiographic and systematic assessment method, which can be used to monitor the top three youth problems on a session-by-session basis. Further, new technologies such as iPods, smart phones, or other electronic devices are youth-friendly manners to help them monitor their complaints on such regular levels.

### Treatment Conditions

With regard to treatment conditions in youth mediation studies, there are at least five possibilities. The first one is to have no treatment comparison condition; the experimental condition is the only treatment of interest, and changes in the mediator during this treatment are investigated. The problem with investigating mediators in single treatment designs is that no evidence can be found that changes in the mediator are caused by the active treatment and not by other factors (such as passage of time). But sometimes, practical considerations hinder the use of a comparison condition, and ethical considerations can often be the reason why random assignment to a wait list condition is not feasible. In this case, several ways are proposed in which a stronger evidence for mediation can be achieved. First, it is better not to use statistical approaches that rely on between treatment-differences (i.e., Baron and Kenny 1986; Kraemer et al. 2002). The studies by Hogendoorn et al. (2011) and Maric et al. (2011) illustrate the use of alternative statistical approaches (e.g., MacKinnon 2008) for single-condition designs. Second, a potential non-mediating variable, which is assumed not to be affected by the active treatment, can be included (MacKinnon 2008). A third alternative is to examine the influence of treatment dosage (e.g., 4 vs. 8 sessions of problem-solving training) on the mediator (e.g., self-control) and to investigate whether greater dosages lead to greater changes in the mediator which, in turn, lead to beneficial treatment outcome (Stice et al. 2010). Still, despite these possibilities, in the absence of a treatment control group, alternative explanations for the mediating effects cannot be ruled out.

A stronger test of mediation is achieved when a control condition is included in the design. Thus, a second possibility for a treatment comparison condition is to include a waitlist control condition. To date, several treatment outcome studies with youth have included a waitlist control condition (e.g., Beauchaine et al. 2005; Kendall and Treadwell 2007; Treadwell and Kendall 1996). Beauchaine et al. found less child externalizing problems at follow-up when more treatment components (i.e., child, parent, teacher) were delivered to families. In the waitlist group, no mediating effects were found on any of the proposed mediators (parenting and child behavior, treatment dosage). Kendall et al. found that changes in the proposed mediator (negative self-statements) were associated with changes in anxiety outcomes in the CBT condition, but not in the waitlist control group. A third possibility for a comparison condition is another active treatment. Comparing two or more treatments allows a direct investigation of treatment specific mediators. For example, Hagen et al. (2011) found that effective discipline and family cohesion at posttreatment were mediators of Parent Management Training at follow-up in children with conduct problems, but were not mediators of treatment outcome in the comparison conditions (e.g., family therapy, behavioral therapy, cognitive therapy).

A fourth possibility is to include a treatment comparison condition which is devoid of specific elements of the treatment such as cognitive restructuring and behavioral techniques in the case of CBT, while still providing a supportive environment (Brent et al. 1997; Last et al. 1998). Such a condition would control for the non-specific aspects of the CBT such as therapeutic alliance and therapist’s warmth and attention. Designs comparing CBT and non-specific treatment conditions are very useful to test mediation, because if cognitions were observed to change for those in the CBT condition and not for those in the non-specific condition, and this resulted in decreased anxiety after CBT, then more evidence would be gained for the role of cognitions in mediating CBT outcomes. Further, we would be more certain that specific CBT components led to these changes in cognitions and not, for example, therapeutic alliance. Several studies with anxious youth (Hudson et al. 2009; Silverman et al. 1999) and youth with school refusal (Last et al. 1998) have developed and utilized such a control condition to investigate the efficacy of CBT, but have not taken advantage of this comparative study design to also report on the mediators of CBT outcomes.

All of the above-mentioned designs do not inform us enough about which specific treatment components are effective within a certain treatment. In the case of CBT and youth anxiety, for example, it is still unknown whether cognitive therapy or exposure leads to changes in emotion, cognition, and behavior. In the field of externalizing disorders, Hinshaw et al. (2000) provide a good example of testing mediation using different treatment modalities (i.e., medication management, behavioral treatment, community care) alone and in combination for childhood ADHD. The authors found that the combined pharmacological and behavioral treatment led to decreases in parental ineffective discipline which led to increases in the quality of child’s social functioning at school. This type of dismantling designs helps to shed light on the question which specific treatment components are active in bringing up the change in the mediator which in turn leads to changes in the treatment outcome for youth internalizing and externalizing disorders.

### Experimental Manipulation of the Mediator

To some extent, there is a certain overlap between this paragraph and the previous one as both describe different treatment conditions in the study of mediation. This paragraph, however, extends the previous one by focusing on how treatment conditions can be used to test mediators as causal processes. Even when groups are randomized to different treatment conditions and the assumption of temporal precedence is met, one has to acknowledge that statistical mediation analyses based on nonexperimental data provide inconclusive evidence regarding mediators as causal processes (MacKinnon 2008). More definite conclusions with regard to causality can be drawn in randomized designs that involve direct manipulations of a mediating variable. Before we turn to a few examples, it should be noted that these designs are not always feasible in youth clinical research practice because of practical and ethical considerations, but they are not impossible.

The first example concerns a blockage design that uses an experimental manipulation to block the mediation process (MacKinnon 2008). If the resulting mediation relation is being removed, there is evidence for the mediation process. For example, consider a study that investigates the extent to which a treatment reduces anxiety by changing dysfunctional cognitions through cognitive therapy techniques. Participants receiving the treatment may be randomly assigned to a condition in which cognitive therapy techniques were eliminated or to a control condition that allowed cognitive therapy techniques. If changes in dysfunctional cognitions are a mediator of anxiety treatment, then reduced levels of dysfunctional cognitions should be observed in the control condition, but not in the experimental condition. Non-specific treatment conditions such as mentioned in the previous paragraph may be used as a control condition in these types of designs.

A second type of design used to manipulate the mediator is called enhancement design (MacKinnon 2008). This design is similar to the blockage design, but elevated levels of the mediator are used to enhance the mediation process. For example, consider a study that investigates the extent to which a treatment reduces depression by changing physical activity through behavioral activation. An enhancement design would randomly assign youth with depression to groups with varying levels of physical activity. If behavioral activation treatment reduces depression through physical activity, the largest beneficial effects should be observed in groups randomly assigned to receive the highest dose of physical exercise.

On a single-case level, experimental manipulation of the mediator can be achieved through a (sequence of) treatment introduction and withdrawal design (Barlow et al. 2009). Although this design sounds attractive from an experimental point of view, from the clinical perspective, it may be undesirable (i.e., consider withdrawing treatment to a young depressive client). However, despite this limitation, this design can be a useful research tool when natural factors such as therapist’s or client’s vacation or illness interfere.

Use of an experimental design with careful manipulation of the potential mediator is essential for demonstrating causality. In experimental psychopathology, a recent line of research is primarily aimed at directly manipulating cognitive variables hypothesized to play a role in psychopathology, that is research on cognitive bias modification (CBM). For example, the seminal study by MacLeod et al. (2002) reported that an attentional bias for threat-related stimuli can influence subsequent emotional vulnerability, in either direction: participants with medium anxiety levels who were trained toward threat stimuli were more easily stressed, while participants who were trained away from threat stimuli were less easily stressed in a subsequent stressful task. Obviously, the second manipulation has more clinical ramifications, and in clinical groups, usually a control group receives no modification (see Wiers et al. 2006 for CBM-designs). Several studies have now demonstrated positive results of CBM in clinical samples of adults (e.g., in social anxiety, Amir et al. 2009; Schmidt et al. 2009) and in addiction (Schoenmakers et al. 2010; Wiers et al. 2011). However, it should be noted that in many of these studies, no significant mediation was reported (most likely related to measurement problems with the mediator, MacLeod et al. 2009), and clinical effects have been modest (Bar-Haim 2011; Hallion and Ruscio 2011). As yet, very few studies have applied CBM to children and adolescents, but there are some promising examples (Rozenman et al. 2011; Salemink and Wiers 2011).

### Single-Case Experimental Designs

Single-case experimental designs (SCEDs) are reliving their comeback as a powerful tool that can be used to examine the efficacy and effectiveness of youth treatments (Barlow et al. 2009). Although it suggests that the focus of the investigation is on a single person, the term actually refers to the level of statistical analysis. The often-noted problem of generalization can be resolved through several replications. Back in 1995, the Task Force of the Society of Clinical Psychology, a division of the American Psychological Association, classified interventions tested in nine replicated single-case studies as ‘well-established’.

There are at least two reasons why single-case experimental designs are useful for examining mediators of youth treatment outcomes. First, the mediators of newly developed treatments can be investigated on a small scale prior to investigations in randomized clinical trials (Norell-Clarke et al. 2011). Second, single-case experimental designs involve the possibility to capture multiple mechanisms (i.e., child, parent, family, school) which are likely to be related to each young client’s functioning (MacKinnon 2008). This is especially relevant for heterogeneous populations of youths (Gaynor and Harris 2008; Maric et al. 2011) and for treatment packages that include several treatment components. Otherwise, the effects of mediators may be lost when mediation analysis is being conducted on a group-level only. In their study on the effects of behavioral activation intervention for depression in adolescence, Gaynor and Harris (2008) described a strategy for testing the mediators of treatment outcome in four adolescents with depression. Potential mediators (i.e., automatic thoughts, coping, engagement in pleasant activities) and treatment outcome variable (depression) were assessed on pre-, post-, and follow-up, and prior to each session. For two adolescents, significant clinical improvement during the active phase of the treatment (i.e., behavioral activation) was mediated by increases in engagement in pleasant activities. That is, the patterns of change, both graphically and statistically, showed that the increases in activity levels temporally preceded decreases in adolescent depression. The authors were able to conclude that in 50% of the cases, increased activation was shown to be an important mediator of treatment outcome for adolescent depression. Another example by Maric et al. (2012) concerns an ongoing study on the mediators of exposure and cognitive therapy in adolescents with anxiety disorders. Two adolescents with anxiety disorder receive 4 weeks of exposure only, and 4 weeks of exposure plus cognitive therapy. Assessments of potential mediators and treatment outcome variables (coping, anxiety, negative and positive automatic thoughts, and avoidance behavior) are conducted during baseline, at pre-, post- and follow-up, and regularly during treatment. An idiosyncratic approach to assessment is used, meaning that prior to each session the top three items (i.e., highest scores of the client) from measures are taken. On a daily level, assessments of anxiety and coping are also conducted. Youth-friendly methods have been used such as internet and mobile phones to facilitate completion of assessments. Regarding data-analytic techniques, time-series analyses such as those described by Barlow et al. (2009) are used. Besides examining the incremental value of cognitive therapy above exposure, this design allows us to investigate: (a) mediators of exposure (coping, anxiety, negative, and positive automatic thoughts, and avoidance behavior); (b) reciprocal mediation models (e.g., does change in negative cognition leads to change in anxiety or vice versa); and (c) sequential mediation models (e.g., does change in negative cognition lead to change in anxiety which leads to changes in avoidance behavior?). We come back to sequential and reciprocal mediation models in Way 10.

### Statistical Approach

Statistical challenges have been identified as one of the most important obstacles to the study of mediation in youth treatment outcome research (Holmbeck 1997; Kraemer et al. 2002, 2007; Weersing and Weisz 2002). Most researchers are familiar with the Baron and Kenny (1986) article that presented an important statistical approach for the investigation of mediation. It is the most common approach to study mediation in the psychological literature in general, and in the youth treatment outcome studies in particular. According to this approach, four conditions need to be met when investigating the mediation of treatment outcome: (1) treatment needs to effect the treatment outcome (path *c* in Fig. 1, full line), (2) treatment condition should predict changes in the mediator (path *a* in Fig. 1), (3) while controlling for the treatment, change in the mediator should be significantly associated with change in the treatment outcome (path *b* in Fig. 1), and (4) when change in the mediator is statistically controlled for, the effect of treatment on change in treatment outcome is attenuated (path *c’* in Fig. 1, dashed line^1^).

In a simulation study, MacKinnon et al. (2002) compared several statistical approaches to mediation. Their results suggested that the Baron and Kenny method has low Type I error rates and low statistical power in studies with relatively small sample sizes (e.g., *N* ≤ 50). Hence, chances are that no mediation relation will be found, unless the effect or sample size is large. MacKinnon et al. further found that the most important conditions for mediation are that the ‘a’ coefficient is statistically significant [condition (2), Baron and Kenny approach] and that the ‘b’ coefficient [condition (3), Baron and Kenny approach] is statistically significant, based on Type 1 error rates and statistical power. As a result, only conditions (2) and (3) are required to establish mediation. In this so-called product of coefficient test, the product of coefficients from the independent variable to the mediator (*a* path in Fig. 1) and the coefficient from the mediator to the dependant variable adjusted for the independent variable (*b* path in Fig. 1) is divided by the standard error of the product to create a test statistic. This test statistic is then compared against a normal distribution to test for significance. The conclusions of MacKinnon et al. (2002) are important, especially in light of several studies (i.e., Kazdin and Wassell 1999; Kolko et al. 2000) that did not continue with further mediation analyses because of the non-significant first step (path *c*) of the Baron and Kenny analyses. In the product of coefficient approach, the use of conditions (1) and (4) of the Baron and Kenny method may still be of help with regard to the interpretation of the mediating effect, whether the mediation is partial or total.

The MacArthur approach (Kraemer et al. 2002; Kraemer et al. 2008) for the investigation of mediators of treatment outcome deserves attention in this paragraph. The conceptual basis of this approach is the same as in Baron and Kenny’s approach, but the operational framework differs in several ways from it. For example, in the MacArthur approach, the focus is on demonstrating temporal precedence which is required to establish mediation (i.e., mediator occurs during the treatment as a consequence of treatment, and prior to treatment outcome), and a mediator must be correlated to the treatment (Kraemer et al. 2002). Thus, in this approach, there is a strict requirement of measuring a mediator before treatment outcome. There are some conceptual difficulties related to this. If a mediator must occur prior to treatment outcome, then mediators in cross-sectional models and in half-longitudinal ‘contemporaneous’ models (i.e., pre-post-treatment; Cole and Maxwell 2003) could not be investigated. Despite limitations, the MacArthur approach is a clear way to organize analyses of mediation variables in randomized clinical trials (Kraemer et al. 2002).

Often, when choosing the statistical method to study mediation in youth treatment outcome studies, concerns are being raised regarding the required sample size. As mentioned above, the simulation study by MacKinnon et al. (2002) showed that the Baron and Kenny approach has low power and therefore requires a large sample size. From a more recent simulation study by Fritz and MacKinnon (2007), it appeared that as the effect of the direct *c* path (treatment → outcomes) decreased, the Baron and Kenny method required a larger sample sizes, going up to over 20,000 participants for a complete mediation model (*c* = 0), when effects of *a* and *b* paths are small, and for the power of .8 to be achieved. Using a similar simulation methodology, MacKinnon (2008) further showed that, under the same conditions, the product of coefficients test and asymmetric confidence limits method requires over 500 participants. When paths *a* and *b* are large, the latter method would require approximately 33 participants to find a mediating effect with .8 power, while the Baron and Kenny method would need approximate 92 participants to find the same mediating effect. These findings are especially relevant for youth psychotherapy research since small samples are common. For example, Siqueland et al. (2005) investigated the feasibility, acceptability, and efficacy of CBT and attachment-based family therapy in a sample of 11 adolescents with anxiety, and Bögels and Siqueland (2006) investigated the efficacy of family CBT in a sample of 17 children and adolescents with anxiety, and their families. At least in the latter study, mediation analysis could have been conducted with reasonable statistical power, using modern methods as advised by MacKinnon (2008).

In their 2004 article, MacKinnon et al. concluded that besides product of coefficients test, bias-corrected bootstrap had accurate Type I errors and greatest power. This method requires a random sample to be taken from the original data with replacement. The values of *a* and *b* paths are then calculated for this new, bootstrap sample, and the indirect effect, *ab*, is calculated. This process is repeated large amount of times (e.g., 1,000 or 10,000 times). The advantage of bias-corrected bootstrap above other types of bootstrap procedures is that it corrects for skew in the population. An example of a bootstrap procedure can be found in Wiers et al. (2005). The authors used bias-corrected bootstrap method to test for the mediational role of explicit and implicit alcohol-related cognitions of an intervention designed to change positive alcohol expectancies among young adult males. Evidence was found for explicit alcohol-related cognitions mediating reductions in alcohol use at 3-week follow-up intervention. Although bias-corrected bootstrap seems to be a useful method to be used in youth treatment outcome studies, product of coefficient test may be more suitable for very small samples and more feasible in terms of programs and steps that are needed to conduct the analyses (i.e., regression in SPSS vs. multiple computations in AMOS or EQS) [MacKinnon et al. 2004].

### Combining Moderators and Mediators

Many (clinical) researchers advocate the evaluation of mediating and moderating effects within one and the same study (Baron and Kenny 1986; Edwards and Lambert 2007; Fairchid and MacKinnon 2009; Kraemer et al. 2002; Preacher et al. 2007; Rose et al. 2004). One might argue that an already complex mediation analysis should not be made even more complicated by combining it with moderation analysis. However, there are at least two important arguments for investigating mediation and moderation effects together. First, within one and the same study, we would be able to answer two questions: (1) how does a certain treatment work; and (2) for whom does the treatment work and who is in need of an alternative treatment? Second, from a statistical point of view, it is also possible that, when only testing treatment mediation, treatment mediators are not discovered because statistical analyses are conducted over the whole group (MacKinnon 2008).

Two general conditions are possible when combining mediating and moderating effects together: (a) moderation of a mediated effect, that is, the mediating effect is different for different values of a moderator (e.g., subgroups of clients) and (b) mediation of a moderated effect, that is, a mediation relation explains a significant interaction (moderation effect) in the data. An example of moderation of a mediated effect is a case in which a mediation process differs for different age groups (i.e., children vs. adolescents). CBT may affect a sense of self-efficacy for both children and adolescents, but an increase in self-efficacy leads to a decrease in anxiety symptoms for adolescents only. An example of mediation of a moderated effect is when an effect of a CBT program depends on certain personality traits, and this interaction changes a mediating variable of negative cognitions, which then affects depressive symptoms. Although statistically similar, it is important to note that moderated mediation and mediated moderation are not equivalent hypotheses when viewed conceptually. The former is based on the notion that an entire mediation model is significant only at certain levels of a moderator. The latter is based on the notion that the overall moderation of the treatment effect is reduced once the mediating process is controlled for (Muller et al. 2005; Rose et al. 2004). Several researchers provide detailed statistical guidelines for these two analyses (Edwards and Lambert 2007; Fairchid and MacKinnon 2009; Muller et al. 2005; Preacher et al. 2007).

From the clinical perspective, a case of moderated mediation is probably the more relevant one, because results give specific directions regarding efficacy of a treatment for certain groups and not for other groups. An example of a moderated mediation investigation is the study by Tein et al. (2004), which tested a preventive intervention for children from divorced families. The results indicated that the observed program effects in reducing posttest internalizing problems were mediated by improvement in mother–child relationship quality. Program effects reducing externalizing problems at posttest and 6 months follow-up were mediated by an improvement in posttest parental methods of discipline and mother–child relationship quality. These mediation effects were found primarily for children who at the beginning of the program had poorer scores on discipline, mother–child relationship quality, and externalizing problems.

### Reciprocal and Sequential Mediation Models

Most mediation studies discussed in this article expect to find straightforward mediation effects of the treatments investigated, that is, treatment *X* will affect mediator M, which will lead to outcome Y. When no evidence is found for mediation effects in straightforward models, this does not necessarily imply that the mediator was not well chosen or that the treatment failed to produce changes in the potential mediator. Two other situations are imaginable when testing mediation of youth treatment outcomes: reciprocal and sequential mediation relations.

In reciprocal mediation models, one is assuming a different order of causal relations, that is, there is a reciprocal causation between the mediator *M* and the dependent variable *Y*, so that both *X* → *M* → *Y* and *X* → *Y* → *M* can be true. For example, using multilevel mediation analysis, Moscovitch et al. (2005) investigated interactive change in social anxiety and depression among adults with social phobia who participated in weekly sessions of cognitive-behavioral group therapy (CBGT). Two models were tested: Time (Session) → social anxiety → depression, and Time (Session) → depression → social anxiety. The results indicated that, although both feelings of anxiety and depression decreased during treatment, decreases in social anxiety fully mediated decreases in depression, while decreases in depression only partially mediated decreases in anxiety. The authors concluded that during CBGT for social anxiety, depression improves over the course of treatment because social anxiety improves. In another, recent example, Hogendoorn et al. (2011) tested whether an increase in perceived control during CBT for youth anxiety preceded or followed a change in anxious feelings. The authors found that changes in parent reported anxiety symptoms preceded an increase in perceived control. In an alternative model, the authors found further indication for a reciprocal effect where an increase in perceived control and decrease in anxiety symptoms influenced each other over time.

In sequential mediation models, two or more mediators intervene in a series between an independent and a dependent variable (*X* → *M*1 → *M*2 → *Y*). An example of a sequential mediation model is when a treatment (e.g., parent management training) changes the first mediator (e.g., effective discipline), this mediator influences changes in the second mediator in the model (e.g., risk behaviors) and this leads to changes in the treatment outcome (e.g., conduct problems). A recent example of sequential mediation is reported in a study on mediators of multisystemic therapy outcomes for adolescents with externalizing problems (Deković et al. 2011). It was hypothesized that changes in parental sense of competence would lead to positive changes in parenting (positive discipline, inept discipline, relationship quality), which in turn, would lead to changes in adolescent externalizing problems. Evidence was found for a sequential pattern of change in which changes in parental sense of competence predicted changes in positive discipline, and this led to a decrease in adolescent externalizing problems. Both models, reciprocal and sequential, should be tested more often in youth treatment outcome research.

## A Scientific Ladder of Mediation Evidence

**Fig. 2:**
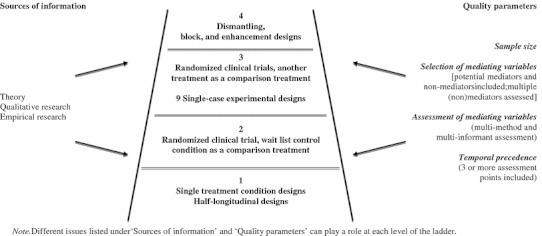
A scientific ladder of mediation evidence

Researchers should aim for the strongest mediation design possible. We do acknowledge that because of practical and/or ethical concerns in the field of youth psychosocial treatments, it will not always be possible to conduct an ideal mediation study. However, the current practice is that mediation analysis is either infrequently reported or that too often suboptimal research designs and methods are used to test mediating relationships. As we have demonstrated in this article, in most research designs, some form of mediation analysis should be possible. At the same time, to be able to draw valid conclusions about the efficacy of our treatments from the mediation studies, we are in need of standards to properly value the findings. This can best be done based on research designs. Mediation studies can be placed at different levels on a ladder of evidence, based on the research design of the study. Studies at every level can contribute to our knowledge and understanding of mediation processes, but the strongest evidence for mediation is provided by studies that are high on the ladder. The weakest evidence for mediation is provided by studies low on the mediation ladder. The strongest evidence is, to a large extent, characterized by the fact that the design provides the researcher with more evidence for a mediator as a causal link between treatment and outcomes. In Fig. 2, a model with four levels of mediation evidence is presented. Indeed, it should be noted that this 4-pt metric does not capture all aspects of mediation in child and adolescent treatment research, but may serve as a heuristic to encourage tests of mediation. As indicated in the column on the left side of the ladder, various sources of information can be used to inform choices for the designs of the mediational studies. For example, choice of potential mediators can be based on theory, qualitative and empirical research, separately or in combination with each other. On the right side of the ladder, different quality parameters are listed related to the needed sample size, the number and type of the potential mediators included, method of assessment and the potential to satisfy the temporal precedence requirement. Generally, the more issues from both sides of the ladder are considered in a mediational study, the more valid conclusions can be drawn about mediation relations and the strength of evidence. However, some caution in the use of quality parameters should be noted. For example, incorporating multiple mediators using insufficient sample sizes can actually reduce the quality of a mediational study instead of enhancing it.

The first level of the mediation ladder represents mediational studies such as by Alfano et al. (2009) that have used an active behavioral treatment condition without a comparison group, and a half-longitudinal design with assessment of mediators and treatment outcome variables at two assessment points (pre- and post-treatment). The second level represents trials in which a randomization process has been used to allocate participants to an active treatment group and a waitlist control condition. Examples of these studies are the ones by Kendall and Treadwell (2007) and Beauchaine et al. (2005). Using the quality parameters, the variance in the mediation ladder can be illustrated with these two studies. While Kendall et al.’s study used a waitlist control group, it only incorporated two assessment points of mediators and treatment outcome variables. Based on the quality parameter of ‘temporal precedence’, this study could be placed lower on the mediation ladder within level 2 than the Beauchaine et al. study. In that study, an active treatment group was compared to a waitlist control group, and three assessment points (pre-, post-, follow-up) of mediators and treatment outcomes were incorporated in the design. Clinical trials that allocate participants to two different treatment groups and studies that replicate findings in nine single-subject experimental designs (SCEDs) may be placed on the third level of the ladder. The study by Kolko et al. (2000) represents an example of comparing two different treatment groups, such as cognitive-behavioral therapy and systemic behavioral family therapy, to investigate mediators of treatments for adolescent depression. A pilot study on the efficacy of EMDR on trauma symptoms in adults with mental disabilities (Dautovic et al. 2011) is a recent initiative in which 9 subjects were being investigated. Extending the design of this study with a control group and regular assessments of mediators could provide a useful design to investigate processes through which EMDR works to improve trauma-related outcomes. Finally, on level four, dismantling, block and enhancement designs can be found. The dismantling design is illustrated in the study by the MTA group (1999) in which different treatment modalities (i.e., medication management, behavioral treatment, community care) were used alone and in combination with each other. Block designs are represented in randomized clinical trials that have compared a CBT condition and a non-specific treatment conditions (e.g., Hudson et al. 2009; Silverman et al. 1999). However, a better design to test for causality would be if the active treatment condition is directed toward manipulation of only one variable instead of a set of variables like in CBT. An example of such a treatment condition is, for example, the previously mentioned cognitive bias modification training procedure. Finally, an example of an enhancement design would be a study in which youths are randomly assigned to groups with varying levels of the mediator. If treatment influences outcomes through the mediator, then the largest benefits should be observed in groups assigned to the highest doses of the mediator.

## Concluding Comments

One of the most important questions raised in youth mental health research today is how treatments for emotional and behavioral disorders in children and adolescents work; what needs to be changed so that better treatment outcomes can be achieved. Current practice does not allow researchers and clinicians to draw valid conclusions regarding evidence for active mechanisms of change in youth treatment. In order to improve upon this practice, we must conduct studies on mediation of youth treatment outcomes using guidelines recommended for mediation analyses. Further, we urge editors and reviewers of manuscripts describing youth treatment outcomes to demand conduct and description of the mediation analyses for the research designs that can be placed on the mediation ladder, so that this analysis can become a common practice in youth treatment outcome research rather than an exception. We do acknowledge that investigating mediation of treatment outcomes is a complex endeavor. Nevertheless, if we want to make progress in this area, both theoretically and clinically, we have to climb the ladder and gain understanding of how and why youth treatments work.

## Footnotes

1 Of course this line can also be a ‘full line’ if there is complete mediation; that is, if all the change in the treatment outcome caused by the treatment happens via the mediator. In treatment studies, this should less often be the case, because of the potential multiple mediators (process variables) occurring before, during, and after the treatment.
